# Study protocol for a randomised controlled trial evaluating the efficacy of a telehealth program – management of asthma with supportive telehealth of respiratory function in pregnancy (MASTERY^©^)

**DOI:** 10.1186/s12890-015-0082-3

**Published:** 2015-07-31

**Authors:** Elida Zairina, Michael J. Abramson, Christine F. McDonald, Jonathan Li, Thanuja Dharmasiri, Kay Stewart, Susan P. Walker, Eldho Paul, Johnson George

**Affiliations:** Centre for Medicine Use and Safety, Monash University, Parkville, VIC Australia; Department of Epidemiology and Preventive Medicine, Monash University, Melbourne, VIC Australia; Allergy, Immunology & Respiratory Medicine, The Alfred Hospital, Melbourne, VIC Australia; Department of Respiratory and Sleep Medicine, The Austin Hospital, Heidelberg, VIC Australia; Department of Electrical and Computer Systems Engineering, Faculty of Engineering, Monash University, Clayton, VIC Australia; Department of Maternal Fetal Medicine, Mercy Hospital for Women, Melbourne, Australia; Department of Obstetrics and Gynecology, University of Melbourne, Victoria, Australia; Department of Clinical Haematology, The Alfred Hospital, Melbourne, VIC Australia

**Keywords:** Asthma, Pregnancy, Telehealth

## Abstract

**Background:**

Telehealth has the potential to improve asthma management through regular monitoring of lung function and/or asthma symptoms by health professionals in conjunction with feedback to patients. Although the benefits of telehealth for improving asthma management have been extensively studied, the feasibility of telehealth for supporting asthma management in pregnant women has not been investigated. This study aims to evaluate the use of telehealth for remotely monitoring lung function and optimising asthma control during pregnancy.

**Methods:**

A randomised controlled trial comparing usual care with a telehealth program (MASTERY^©^) has been conducted. The intervention comprised a mobile application – *Breathe-easy*^*©*^ supported by a Bluetooth-enabled handheld device (COPD-6®), which was used for self-monitoring of lung function (FEV_1_, FEV_6_) twice daily, and recording asthma symptoms and medication usage weekly; and a written asthma action plan (WAAP). The primary outcome measure is change in asthma control measured using the Asthma Control Questionnaire (ACQ). Secondary outcomes include changes in mini-Asthma Quality of Life Questionnaire (mAQLQ) score, lung function, asthma-related health visits, days off work/study, and oral corticosteroid use. Outcome data were collected at baseline, 3 months and 6 months by a research assistant masked to group allocation. Maternal and neonatal outcomes were also collected post-partum.

**Discussion:**

This is the first study to evaluate the application of telehealth to optimize asthma management in pregnant women. If effective, this telehealth program could improve asthma self-management by pregnant women which may reduce the maternal and fetal risks of poorly controlled asthma during pregnancy.

**Trial registration:**

Australian New Zealand Clinical Trials Registry (ACTRN 12613000800729) 17 July 2013

**Electronic supplementary material:**

The online version of this article (doi:10.1186/s12890-015-0082-3) contains supplementary material, which is available to authorized users.

## Background

Asthma is the most common obstructive pulmonary disease that occurs during pregnancy and may complicate pregnancy [1–4]. Previous studies have demonstrated that asthma during pregnancy improves in slightly more than a quarter of patients, but worsens in slightly more than one-third, and remains unchanged in one-third [5]. Poorly controlled asthma increases the risk of poor outcomes such as pre-eclampsia, fetal growth restriction, preterm birth and need for caesarean delivery [6–9]. Given the lifelong health decrements associated with these outcomes, an approach to optimise monitoring and treatment of asthma could have major short and long term public health benefits.

Education and regular monitoring by a pharmacist-led multidisciplinary team has been shown to improve asthma control during pregnancy [10]. Asthma management programs for pregnant women which involve regular monitoring of lung function and assessment of asthma symptoms appear to be effective in reducing asthma exacerbations [11, 12]. However, further studies are required to determine the most effective and safe interventions for managing asthma in pregnancy to improve maternal and neonatal outcomes [12].

Better asthma control can be achieved if patients are involved in self-management. This includes self-monitoring of asthma symptoms or lung function and following written asthma action plans while maintaining regular contact with their health professionals [13]. Telehealth supports asthma management through patient education, adherence support, telephone follow-up and remote monitoring [14]. Telehealth interventions have shown significant clinical improvement and potential benefits in patients with asthma, including: reduced time to access healthcare services and reduced cost linked to travelling; earlier detection of worsening asthma (e.g. exacerbations), and reduced healthcare visits/hospitalisations due to asthma [15–18]. Lung function data derived from telehealth assessment studies were comparable to those collected under supervision by healthcare professionals and thus valid [19, 20]. Telehealth programs also appeared to be feasible for asthma patients, as compliance with monitoring was high and most patients found the equipment easy to use [21].

Telehealth studies show potential benefits for asthma management in the general population [22]. However the feasibility of telehealth for supporting asthma management in pregnant women has not been investigated to date. Pregnant women with asthma are young and are likely to be willing to use new technology to assist self-management of their asthma. Hence, the present study will examine the potential for enhanced asthma management in pregnancy through a telehealth program.

The study aims to evaluate the efficacy of a telehealth intervention supported by a handheld respiratory device and a written asthma action plan (WAAP) for management of asthma in improving asthma control during pregnancy. We hypothesised that the telehealth intervention group will have better asthma control as measured by the Asthma Control Questionnaire (ACQ) score changes than the control group at three and six months from baseline.

## Methods

### Study setting

Participants were recruited when attending scheduled antenatal visits at two large maternity hospitals in Melbourne, Australia (Mercy Hospital for Women and The Royal Women’s Hospital); each hospital has approximately 6000 births per year.

### Study design

**Fig. 1:**
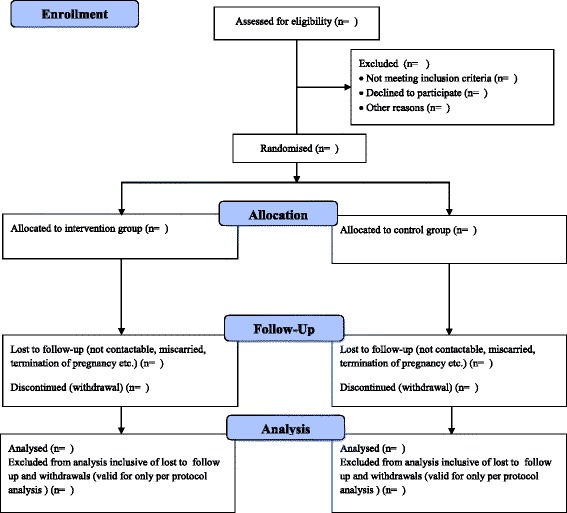
Participant flow diagram

The study was designed as a prospective multi-centre single-blinded randomised controlled trial (RCT) with outcome assessors masked to group allocation at follow-up assessments. The flow of participants is illustrated in Fig. 1. The total duration of the intervention was 6–8 months depending on timing of the first antenatal visit and enrolment in the study. Both groups were followed up throughout pregnancy and the outcomes are being compared at three and six months from baseline to evaluate the efficacy of the intervention.

### Inclusion and exclusion criteria

Eligibility for this trial included pregnant women with asthma who had used any inhaled bronchodilator or anti-inflammatory agent for asthma within the previous 12 months, were attending antenatal clinics in first or second trimester, aged 18 years or older and able to communicate in English. Those under specialist care for severe/difficult asthma were excluded. Women who are not already in possession of or have not used a smart mobile phone were considered ineligible to participate. This was confirmed by directly asking pregnant women about their mobile phone usage.

### Trial recruitment

The following methods of identification and recruitment of participants were used:

1. One of the researchers (EZ) or research staff searched the outpatient files and screened the medical records of pregnant women with asthma scheduled to have a clinic visit on the following day by reviewing GP referral letters and/or notes from previous clinic consultations. Eligible women were approached for recruitment after clinic visit completion. On each day the researcher/research staff also searched for potential participants by screening medical records of women who were reviewed by the midwives or medical staff in the antenatal clinics.
2. At one of the sites (the Mercy Hospital for Women), a letter of invitation including a brief explanation of the study together with their antenatal appointment letter was posted to all pregnant women newly registered with the antenatal clinic. At their first clinic visit, the researcher approached each eligible woman and sought interest in participation.
3. Advertising posters about the study were placed in the antenatal clinics of participating hospitals and on the websites of the National Asthma Council and the Asthma Foundation of Victoria. Participant explanatory statements and expression of interest forms were also made available. Potential participants had access to the information and were asked to leave their contact details to allow one of the researchers to contact them.

If the woman agreed to participate, written informed consent was sought.

### Group allocation

Recruited participants were allocated to intervention or control groups on a 1:1 basis, stratified by their asthma severity. Asthma severity was assessed in accordance with the National Asthma Council Australia (NAC) Asthma Handbook classification based on their current asthma medications and symptoms [23]. Participants were classified into two groups: 1) Intermittent – mild asthma: using relievers only (e.g. salbutamol); 2) moderate – severe asthma: using any regular inhaled corticosteroids/ preventers/ symptom controllers or their combinations.

Allocation was concealed using the sealed opaque envelope technique. Random blocks of four and six were chosen and random numbers were generated using a random allocation software program [24] by an external researcher not involved in the study. The allocation sequence was known to this researcher only. At the time of recruitment, the investigator (EZ) opened the numbered envelope and allocated each participant to the control (usual care) group or the intervention (MASTERY) group. The outcome assessors were masked to the participant group allocation at follow-up assessments.

### Control and intervention group

#### Intervention: MASTERY group

The trial evaluated an intervention involving a telehealth program supported by a Bluetooth-enabled handheld spirometer COPD-6® (model number 4000, Vitalograph Ltd, Ennis, Ireland) and a WAAP. Women allocated to the intervention group were provided with a COPD-6® and a loaned smart mobile phone with the specifically designed *Breathe-easy*^©^ application installed on it. Each participant measured their lung function (FEV_1_ and FEV_6_) daily using the COPD-6® device and recorded asthma symptoms and asthma medication usage in the *Breathe-easy*^©^ application weekly. The daily lung function data were uploaded to a central server where the researchers, participants and their health professionals had secure access to the data. The participants’ health professionals were contacted by one of the researchers, a trained asthma educator (EZ), if any medication changes or unscheduled asthma-related visits were needed.

**Fig 2:**
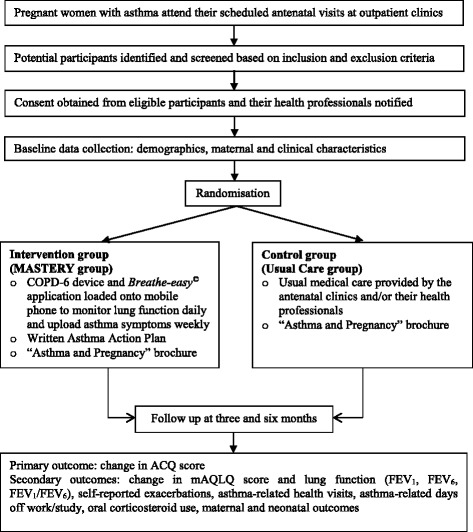
Flow chart of the study

**Fig 3:**
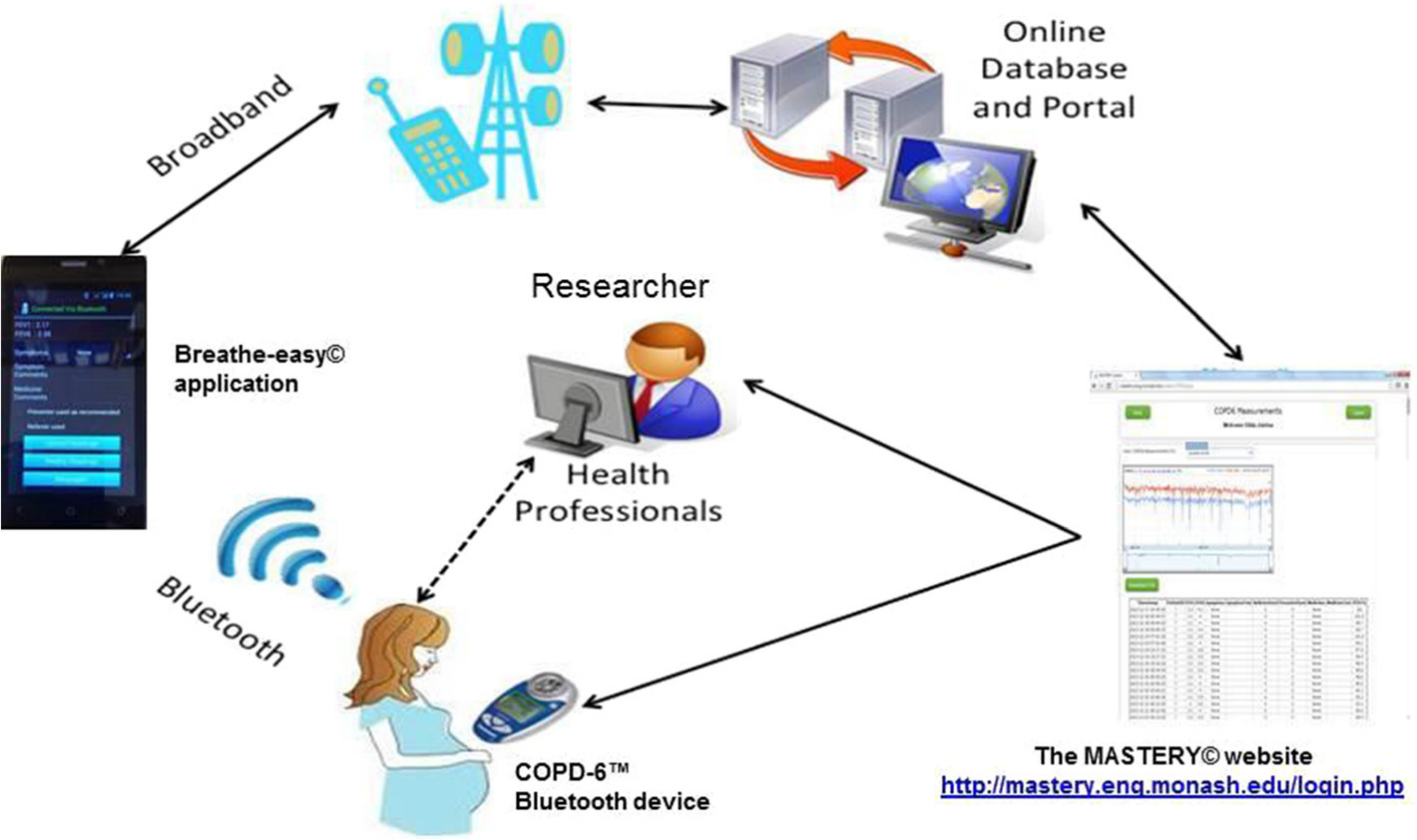
The MASTERY^©^ trial design

A WAAP consistent with NAC guidelines was designed for each participant based on information obtained at baseline. The WAAP contained instructions on which medications to take when feeling well, how to recognise worsening asthma, what to do when symptoms are getting worse and what to do in the event of an acute attack, including a first aid plan. Each participant received an automated weekly message regarding their asthma status based on the *Breathe-easy*^©^ algorithm that was designed based on NAC [23] and Global Initiative for Asthma (GINA) guidelines [25] (Additional file 1: Table S1). An automated weekly message of overall asthma control status was displayed as ‘well-controlled’ (score 0, green zone), or ‘not well controlled’ (score 5, yellow zone and score 6, orange zone) to encourage participants to follow their agreed asthma action plan and/or contact their health professional the next working day if there was no improvement. If the asthma control status was displayed as ‘very poorly controlled’ (score 7–15, red zone), patients were prompted to follow their agreed asthma action plan and contact their health professional on the same day. The flow of the study is described in Fig. 2 and the details of the intervention are illustrated in Fig. 3.

#### Control: usual care group

Women allocated to the control group received the usual medical care provided by the antenatal clinics and/or their health professionals. This included their regular weekly to monthly antenatal visits depending on their trimester and other complications. If during follow up, it was apparent that their asthma control deteriorated since prior assessment (for example; using their reliever three or more times a week, needing to increase their preventer dose), the participant was advised by the research team to contact their health professionals. The control group was also given a summarised version of the “Asthma and Pregnancy” brochure from the NAC which explained about asthma in pregnancy including first aid and emergency assistance number to use for any concerns regarding their asthma.

### Outcome measures

The primary outcome measure was change in asthma control as measured by the Juniper Asthma Control Questionnaire (ACQ - 7) [26]. Secondary outcomes included changes in Juniper’s mini Asthma Quality of Life Questionnaire (mAQLQ) score [27], lung function (FEV_1_ and FEV_6_), self-reported exacerbations (symptoms requiring bronchodilators), asthma-related health visits, days off work/study related to asthma, and oral corticosteroid use. Lung function testing (pre-bronchodilator) was performed by trained research assistants using EasyOne™ Worldspirometer (ndd Medizintechnik AG, Zurich, Switzerland). Maternal and neonatal outcomes data collected are the gestational age of the baby at delivery, development of any antenatal complications such as gestational diabetes, hypertensive disorders of pregnancy, fetal growth restriction, antepartum hemorrhage, and delivery details. Neonatal outcomes collected at delivery include birth weight centile, birth length, head circumference, and Appearance Pulse Grimace Activity and Respiratory (APGAR) scores at 1 and 5 min after delivery. Birth weight centiles were calculated using www.gestation.net/grow-au.aspx, which adjusted for maternal characteristics including height, weight, ethnicity, parity and fetal gender.

### Data collection and follow-up

ACQ, mAQLQ scores, asthma-related health visits, asthma-related days off work/study, and oral corticosteroid use were collected at baseline, 3 months and 6 months from baseline to allow comparisons. Identical data collection forms were used for both groups. Maternal and neonatal outcomes data were collected shortly after delivery by reviewing medical records. The assessors responsible for collecting outcome data at three and six months were masked to the participant group allocation.

### Sample size

A sample size of 28 per arm is sufficient to detect the minimum clinically important difference in ACQ score of 0.5 or more between treatment groups using a standard deviation of 0.66 [10, 28, 29]. We estimate, conservatively, that by 3 months, there will be an improvement of at least 0.5 in the ACQ score in the intervention arm and that the control arm could exhibit a very small improvement or no improvement at all in the ACQ score. If these improvements are sustained at 6 months, then with 28 evaluable subjects in each arm, and assuming an independence model for measurement variation, and an intraclass correlation of 0.5 (equivalent to a within patient variance of 0.218 when the total variance is 0.66^2^ = 0.436), the F-test, conducted at the 5 % significance level will have at least 80 % power to detect a treatment by time interaction effect. If these conjectured improvements by month 3 are not durable and the scores return, on average, to their baseline levels at 6 months, then both the F-test for the interaction and the two-sided *t*-test comparing the two arms at month 3 at the 5 % level of significance will continue to have at least 80 % power. To allow for approximately 25 % attrition, the target sample size has been inflated from 28 per arm to 36 participants per arm. The study is, however, not powered to detect differences in the secondary outcomes.

### Data analysis

All analyses will be performed using SAS version 9.4 (SAS Institute, Cary, NC, USA) and SPSS version 19.0 (IBM SPSS Statistics for Windows, Armonk, NY). The baseline characteristics of the two groups will be compared using Student’s *t*-test for normally distributed continuous variables, Mann-Whitney ‘U’ test for non-normally distributed continuous variables and chi-square or Fisher’s exact test as appropriate for categorical variables. The primary analysis will be performed according to the intention to treat (ITT) principle on the “full analysis set”.

Primary inferential analyses will be conducted using a mixed effects model for the ITT population. This model will include treatment group and time as fixed effects with an interaction between treatment and time to ascertain if the groups behave differently over time. All observed data will be included in the analysis, with the mixed-effects models, fitted by residual maximum likelihood (REML) assuming non-informative dropout such that the probability of dropout may depend on a participant’s previous response but not on current or future responses. In supportive analyses, changes in the primary outcome from baseline to 3 and 6 months will be compared between groups using linear regression modelling adjusting for baseline scores. Baseline demographic and clinical factors that appear to be different will be included as potential covariates in all regression models.

We will also compare the proportion of participants whose ACQ score improves more than 0.5 (MCID) over the study period, the proportion in whom asthma remained “not well controlled’ (ACQ score 1.5 or greater) and those whose asthma was “well controlled” (ACQ score less than 1.5) at each time point [26]. Secondary outcomes will be summarised using descriptive statistics and analyses will be performed using the methods described above.

### Ethical aspects

The study has been approved by the human research ethics committees of Monash University, Mercy Hospital for Women and The Royal Women’s Hospital. All participants provide written informed consent at the time of enrolment. The study has been registered with the Australian New Zealand Clinical Trials Registry: ACTRN12613000800729.

## Discussion

Innovative solutions are needed to support self-management and to improve asthma control during pregnancy. The MASTERY trial is designed to evaluate a telehealth program aimed at helping pregnant women with asthma to monitor their lung function and better manage their asthma during pregnancy. The Vitalograph COPD-6® is designed to enable the lung function data to be transmitted automatically via a Bluetooth connection to another enabled device (e.g. phone, computer). In our study, the Breathe-easy^©^ application installed on a smart phone acquires the data and transmits via internet to a secure website, which can be accessed only by patients and other authorized personnel.

Automatic transmission can minimise the risk of errors when data are entered/reported manually. The *Breathe-easy*^©^ application has been developed to record lung function data, asthma symptoms and medication usage and provides a user-friendly interface through a smart mobile phone. Recording symptoms as part of asthma management is known to reduce costs associated with unplanned hospitalisation and improved quality of life in asthma patients [13, 27, 28].

Daily recording of lung function and weekly assessment of asthma symptoms could allow women to recognize early worsening of their asthma control. Participants in the intervention group were provided with a WAAP as it was part of the algorithm of the telehealth intervention (MASTERY). The automated messages sent to each participant in the intervention group contained feedback regarding their asthma control/condition and asked them to refer to their individualised WAAP for further management. The individualised WAAP designed for each woman provided clear guidelines in terms of actions to be taken in case of worsening asthma. Although the WAAP alone may be effective in achieving asthma control, the telehealth (Breathe-easy^©^) app had additional features for monitoring and recording symptoms and lung function to give instant feedback to participants about their asthma status using an algorithm based on an individualized WAAP.

The proposed intervention has the potential to identify worsening asthma control early and prevent asthma exacerbations during pregnancy by regularly monitoring lung function and asthma symptoms. This may translated to reduce health care costs through fewer asthma-related unplanned medical and emergency department visits. If the intervention is efficacious, this could potentially influence clinical practice and health policy. The *Breathe-easy*^©^ application could be made widely applicable for routine clinical use, particularly for those with chronic respiratory conditions.

### Trial status

At the time of manuscript submission, participant recruitment, randomisation and follow-ups have been completed. The data analysis is currently in progress.

## Additional file

Additional file 1: Table S1.
**The** ***Breathe-easy*** ^©^ **algorithm.**

## Abbreviations

ACQ: Asthma control questionnaire
APGAR: Activity pulse grimace appearance respiration
BMI: Body mass index
FEV_1_: Forced expiratory volume in 1 second
FEV_6_: Forced expiratory volume in 6 seconds
ICS: Inhaled corticosteroid
LABA: Long-acting beta agonist
mAQLQ: mini Asthma Quality of Life Questionnaire
MCID: Minimal clinically important difference
SABA: Short-acting beta2 agonist
