# Supplementary material for: Study protocol for a randomised controlled trial evaluating the efficacy of a telehealth program – management of asthma with supportive telehealth of respiratory function in pregnancy (MASTERY©)
Source: BMC Pulm Med. 2015 Jul 31;15:84. doi: 10.1186/s12890-015-0082-3 (PMC4521452; doi:10.1186/s12890-015-0082-3)
Supplement: Additional file 1: Table S1. — The Breathe-easy © algorithm. [file 12890_2015_82_MOESM1_ESM.pdf]

Table 1 The *Breathe-easy*© algorithm

Symptoms	Night-time awakening	Interference with normal activity	SABA for symptom control (not prevention of exercise-induced bronchoconstriction	FEV$_1$	Total Score	Asthma Control Zone	Levels of asthma Control
≤2days/week **(0)**	0/week **(0)**	None **(0)**	≤2 days/week **(0)**	>80% predicted/ personal best **(0)**	**0**	**Green**	**Well-controlled**
>2 days/week **(1)**	1-3x/week **(1)**	Some limitation **(1)**	>2 days/week **(1)**	70-80% predicted/ personal best **(1)**	**5**	**Yellow**	**Not well-controlled**
>2 days/week **(1)**	1-3x/week **(1)**	Some limitation **(1)**	>2 days/week **(1)**	60-69% predicted/ personal best **(2)**	**6**	**Orange**	
Throughout the day **(3)**	≥4x/week **(3)**	Extremely limited **(3)**	Several times per day **(3)**	<60% predicted/ personal best **(3)**	**7 - 15**	**Red**	**Very poorly controlled**

**Categories**		**Message/Feedback**
**0**	***Well-controlled***	Your asthma seems to be well-controlled. Continue your current asthma management including any medications you are currently using.
**1-5**	***Not well-controlled***	Your asthma seems to be getting worse, refer to your asthma action plan and make any changes in management as recommended.
**6**		Your asthma seems to be getting worse, refer to your asthma action plan and make any changes in management as recommended. If still no improvement, your asthma needs to be reviewed by a health professional.
**7 - 15**	***Very Poorly Controlled***	Follow the Asthma First Aid Plan and call an Ambulance if there is no improvement in your symptoms. Your asthma needs to be reviewed by a health professional.
